# Development of a *Drosophila melanogaster* spliceosensor system for *in vivo* high-throughput screening in myotonic dystrophy type 1

**DOI:** 10.1242/dmm.016592

**Published:** 2014-09-19

**Authors:** Irma García-Alcover, Jordi Colonques-Bellmunt, Raquel Garijo, José R. Tormo, Rubén Artero, Mari Carmen Álvarez-Abril, Arturo López Castel, Manuel Pérez-Alonso

**Affiliations:** 1 Valentia BioPharma, Scientific Park of the University of Valencia, Paterna, Valencia 46980, Spain.; 2 Department of Genetics, University of Valencia, Burjassot, Valencia 46010, Spain.; 3 INCLIVA Health Research Institute, Valencia 46010, Spain.

**Keywords:** Myotonic dystrophy, Splicing, Luciferase, *In vivo* screening, Minigene

## Abstract

Alternative splicing of pre-mRNAs is an important mechanism that regulates cellular function in higher eukaryotes. A growing number of human genetic diseases involve splicing defects that are directly connected to their pathology. In myotonic dystrophy type 1 (DM1), several clinical manifestations have been proposed to be the consequence of tissue-specific missplicing of numerous genes. These events are triggered by an RNA gain-of-function and resultant deregulation of specific RNA-binding factors, such as the nuclear sequestration of muscleblind-like family factors (MBNL1–MBNL3). Thus, the identification of chemical modulators of splicing events could lead to the development of the first valid therapy for DM1 patients. To this end, we have generated and validated transgenic flies that contain a luciferase-reporter-based system that is coupled to the expression of MBNL1-reliant splicing (spliceosensor flies), to assess events that are deregulated in DM1 patients in a relevant disease tissue. We then developed an innovative 96-well plate screening platform to carry out *in vivo* high-throughput pharmacological screening (HTS) with the spliceosensor model. After a large-scale evaluation (>16,000 chemical entities), several reliable splicing modulators (hits) were identified. Hit validation steps recognized separate DM1-linked therapeutic traits for some of the hits, which corroborated the feasibility of the approach described herein to reveal promising drug candidates to correct missplicing in DM1. This powerful *Drosophila*-based screening tool might also be applied in other disease models displaying abnormal alternative splicing, thus offering myriad uses in drug discovery.

## INTRODUCTION

At least 90% of human genes undergo alternative splicing, which generates the high diversity of proteins encoded in the genome (Wang and Burge, 2008). Alterations in splicing are linked to human genetic diseases, resulting in the generation of functional splice variants that are not normally expressed. As a consequence, the correction of disease-associated aberrant splicing events is growing in momentum as a therapeutic approach (Havens et al., 2013; Singh and Cooper, 2012). Advances in understanding splicing regulation, and in the development of large-scale pharmacological screening, have both recently been facilitated by the introduction of minigene fluorescent or luminescent reporter systems (Younis et al., 2010; Arslan et al., 2013; Zheng et al., 2013). It is important to note that many alternative splicing events display precise tissue and developmental regulation, underlining the requirement for the correct biological context when examining functional and therapeutic insights into alternative splicing (Takeuchi et al., 2010; Calarco et al., 2011). That notwithstanding, the greatest use of alternative splicing reporter systems is in cell-based approaches (Younis et al., 2010; Arslan et al., 2013; Zheng et al., 2013), a limitation which can be overcome through the exploitation of *Drosophila melanogaster*, a model organism that simultaneously offers a fully integrated biological system, human-disease-mirroring capabilities and the suitability to assay miniaturization and automation (Pandey and Nichols, 2011).

Myotonic dystrophy type 1 (DM1, OMIM no. 160900) is a neuromuscular disorder linked to a major misregulation of alternative splicing and is considered to be the first described spliceopathy (Udd and Krahe, 2012). DM1 is caused by the expansion of a CTG trinucleotide repeat tract located in the 3′ untranslated region (UTR) of the dystrophia myotonica-protein kinase (*DMPK*) gene. The main pathogenic effect in DM1 is a deleterious gain-of-function of the mutant expanded CUG-containing mRNA (CUG-RNA), which triggers the biochemical and clinical features of DM1. The current model of disease progression derives from the strong interaction of expanded CUG-RNA with splicing regulators such as the muscleblind-like proteins (MBNL1–MBNL3) and CUG-BP Elav-like family member 1 (CELF1), key proteins involved in DM1 pathophysiology (Udd and Krahe, 2012). Importantly, MBNL1 is sequestered by the expanded CUG-RNA in anomalous ribonuclear aggregates (foci), which causes the deregulation of alternative splicing in a large group of pre-mRNAs (Udd and Krahe, 2012). The *muscleblind* gene (*mbl*) is not only conserved in the *Drosophila* genome, but it also plays a role in alternative splicing in this organism, suggesting the conservation of key disease pathways in *Drosophila* (Begemann et al., 1997; Vicente et al., 2007; Machuca-Tzili et al., 2006). This was confirmed by the successful reproduction of tissue-specific DM1 hallmarks such as nuclear foci formation, muscleblind sequestration, missplicing, muscle atrophy and reduced lifespan in flies expressing a disease-associated CTG repeat tract [UAS-i(CTG)_480_ flies] (García-López et al., 2008).

**RESOURCE IMPACT**

**Background**

Myotonic dystrophy type 1 (DM1) is a neuromuscular disorder, which was the first recognized genetic disease described as a spliceopathy – a defective regulation of mRNA alternative splicing – owing to the expression of developmentally inappropriate splice products in particular tissues. In this disease, the mutant mRNAs (which contain expanded CUG trinucleotide repeats, and are called CUG-RNAs) are thought to interact with, and deregulate, splicing modulators, such as the muscleblind-like protein 1 (MBNL1), driving the main pathogenic effect in DM1. A recurrent method for pharmacological screening in human disease, also attempted for DM1, exploits cell-based systems to identify small molecules able to modulate disease-linked splicing alterations. The main limitation of traditional *in vitro* drug discovery approaches is that only a small number of compounds are generally confirmed in further *in vivo* studies, typically owing to inefficacy and toxicity issues. The use of a whole organism, such as *Drosophila*, in the early stages of drug development introduces a higher predictive value for clinical outcome. At the same time, a *Drosophila*-based system allows one to mirror precise disease-linked splicing events that occur in humans, thanks to the conservation of alternative splicing machinery and skeletal muscle function between flies and mammals. The recent development of fast, cheap high-throughput *Drosophila in vivo* screens, positions the fly as a powerful tool for successful drug development.

**Results**

This study describes the generation of the first *Drosophila* spliceosensor model – transgenic flies in which the expression of human disease-linked splicing variants expressed from minigenes is coupled to the expression of a reporter – and their use in the *in vivo* identification of splicing modulators for DM1. It is demonstrated that the spliceosensor flies robustly respond to the presence of the DM1 mutation and to human MBNL1 complementation, whose loss of function explains most of the splicing alterations shown in this disease. Spliceosensor flies were grown on a 96-well plate, allowing the establishment of an *in vivo* platform for pharmacological screening for compounds that modify the DM1 phenotype. More than 16,000 small molecules were evaluated, with 30 different chemical structures positively identified by changes in the luminescence signal. Confirmatory studies revealed promising drug candidates for DM1 that acted through separate mechanisms of action, such as the reduction of aberrant ribonuclear aggregates (foci in which MBNL1 is sequestered by the expanded CUG-RNAs) or the ability to bind toxic CUG-RNA molecules, which indirectly modified the splicing read-out. Remarkably, the automation and miniaturization of the whole fly system reached in *in vivo* high-throughput screens would allow the routine evaluation of ~1000 compounds weekly.

**Implications and future directions**

The successful use of a spliceosensor fly model for pharmacological screening highlights the potential rewards of generating this type of system for drug development in DM1. As key physiological processes are well conserved from flies to humans, the spliceosensor approach could be adapted to screen for compounds that target other types of human splicing alterations. In addition, this system might be used for other diseases associated with molecular changes that could be read through coupled luminescent reporter systems.

As a group, we are interested in the development of original *in vivo* screening approaches that expedite the discovery of new drugs applicable to the correction of disease-linked alternative splicing deregulation. To this end, we have generated a DM1 transgenic fly model with a splicing reporter system that is coupled to the reading of a luminescent protein that is expressed in tissues relevant to disease, which we call ‘spliceosensor flies’. These spliceosensor flies have allowed the successful establishment of a *Drosophila*-based high-throughput screening (HTS) system, used here for rapid and accurate large-scale chemical testing in a DM1 model. This flexible system might also be exploited for other human pathologies with anomalous alternative splicing.

## RESULTS

### Generation of *Drosophila* spliceosensors for DM1

**Fig. 1.**
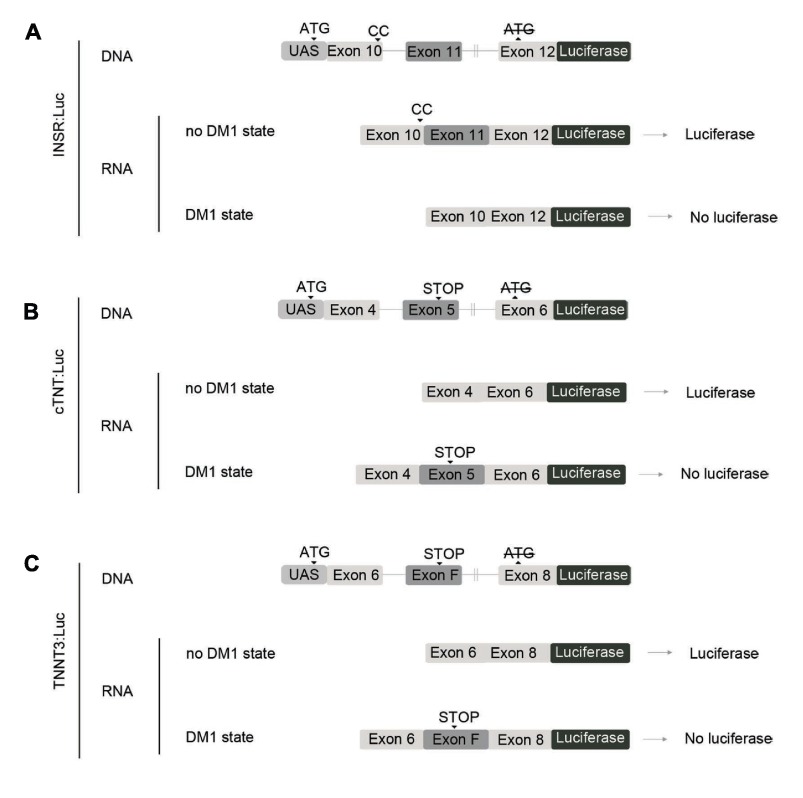
**Generation of DM1 spliceosensor flies.** Schematic representation of *INSR* (A), *cTNT* (B) and *TnnT3* (C) luciferase minigene reporters (INSR:Luc, cTNT:Luc and TnnT3:Luc) injected into *y^1^w^1118^* embryos. In humans, the DM1 state favors *INSR* exon 11 skipping, and *cTNT* exon 5 and *TnnT3* fetal exon inclusion. Each mammalian sequence was cloned in frame with the firefly luciferase ORF (no DM1 state), whose initiation codon was removed (crossed out ATG label) and introduced before each minigene (ATG label). For the INSR:Luc construct, two additional CC nucleotides (CC) were inserted in exon 10 for this purpose. Transgene design envisioned reduced luciferase expression when the CTG repeat expression was induced, as a result of the truncation of its ORF by an exon inclusion (*cTNT* and *TnnT3*) or exclusion (*INSR*). For the cTNT:Luc and TNNT3:Luc constructs, a STOP codon (STOP label) was introduced in the alternative exon so the ORF is truncated in the DM1 state.

The *in vivo* screening platform design started with the generation of the DM1 spliceosensor flies that showed accurate reporter levels correlating with the expressions of the alternative splicing events chosen. Consequently, we adapted previously characterized mammalian minigene constructs that mirror specific alternative missplicing events in DM1 patients. Specifically, we used insulin receptor (*INSR*) exon 11 skipping (Savkur et al., 2001) (Fig. 1A), cardiac troponin (*cTNT*) exon 5 inclusion (Philips et al., 1998) (Fig. 1B), and troponin 3 (*TnnT3*) fetal exon inclusion (Yuan et al., 2007) (Fig. 1C) constructs. To achieve reliable high-throughput automated *in vivo* readouts, we fused the coding sequence of the firefly luciferase gene downstream of each minigene (minigene:Luc) so that luciferase is expressed when the reading frame is unaffected (no DM1 state). Favored alternative splicing events taking place in a DM1 state were designed to either change the reading frame and/or include a premature stop codon, ultimately leading to the absence of the luciferase expression (Fig. 1A–C). The enhancer UAS regulatory region was incorporated upstream of all constructs (UAS-minigene:Luc), thus allowing the use of the UAS-Gal4 system, commonly used in *Drosophila* for the accurate control of transgene expression (Brand and Perrimon, 1993).

**Fig. 2.**
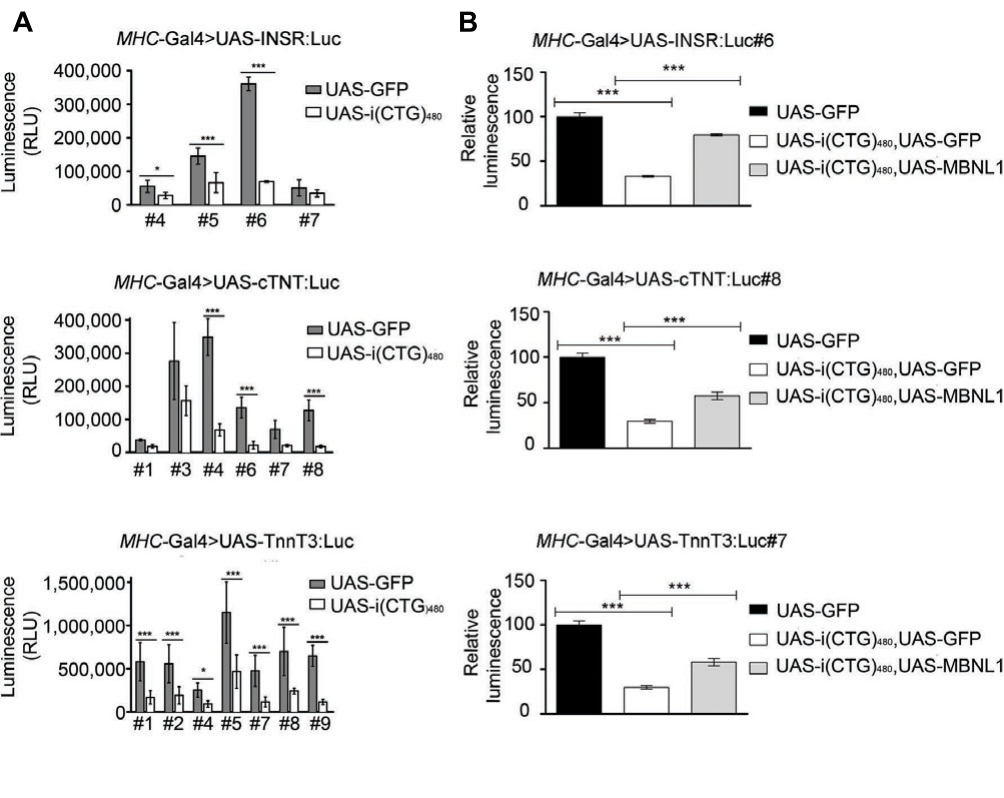
**Characterization of spliceosensor flies.** (A) Luminescence levels of several transformant fly stocks (denoted by the # symbol) for each transgene, when expressed in *Drosophila* musculature with the *MHC*-Gal4 driver, concomitantly with CUG repeat RNA expression [UAS-i(CTG_480_)] (DM1 state, white bars) or with a negative control transgene (UAS-GFP) (no DM1 state, black bars). *P<0.05, ***P<0.001 (Student’s *t*-test). Error bars indicate s.d. (*n*≥6 flies). (B) Luminescence levels of *MHC*-Gal4>UAS-INSR:Luc#6, *MHC*-Gal4>UAS-cTNT:Luc#8 and *MHC*-Gal4>UAS-TnnT3:Luc#7 transgenes in the presence of the CUG repeat RNA expression alone [UAS-i(CTG)_480_, UAS-GFP], and simultaneously with the CUG repeat RNA and human MBNL1 [UAS-i(CTG)_480_, UAS-MBNL1], normalized to the expression in the no DM1 state (UAS-GFP). In all cases, expression of human MBNL1 led to a recovery in luminescence levels. ***P<0.001 (Student’s *t*-test). RLU, relative light units. Error bars indicate s.e.m. (*n*>12 flies). The UAS-GFP is used as a control transgene to balance the number of transgenes in each cross.

Microinjection of *D. melanogaster* embryos with each UAS-minigene:Luc transgene produced several stable transformant lines (Fig. 2A; supplementary material Table S1). We targeted the transgene expression to the musculature of transformant flies by genetic crosses using the *myosin heavy chain* Gal4 driver line (*MHC*-Gal4), which is expressed in somatic muscle. Thus, the transcription of the constructs representing alternative splicing events occurred in a DM1-relevant fly tissue (*MHC*-Gal4>UAS-minigene:Luc), identified as vastly affected in DM1 patients (Udd and Krahe, 2012). The expression of each transgene in the presence of the expanded CUG-RNA [*MHC*-Gal4>UAS-minigene:Luc, UAS-i(CTG)_480_] led to significantly lower luminescence levels in most of the cases, compared with in the absence of toxic RNA (*MHC*-Gal4>UAS-minigene:Luc, UAS-GFP) (Fig. 2A). These results demonstrate the ability of *D. melanogaster* to mirror human DM1 missplicing events.

*MHC*-Gal4>UAS-INSR:Luc#6, *MHC*-Gal4>UAS-cTNT:Luc#8, and *MHC*-Gal4>UAS-TnnT3:Luc#7 were chosen as the best fly transformants based on the identification of the widest window for luminescence levels and the lowest variability after luciferase quantification with and without CTG expression induction (iCTG_480_ flies) (Fig. 2A). In addition, the chromosomal location of the transgenes was taken into account, with intergenic insertion being preferred (supplementary material Table S1). As splicing deregulation has previously been proven to be dependent on MBNL1 in all of the three splicing events that we have exploited (Yuan et al., 2007; Grammatikakis et al., 2011), we assessed whether this splicing factor displayed an *in vivo* ability to rescue luciferase levels in the spliceosensor flies expressing the toxic CUG repeat. Expanded iCTG_480_ and human MBNL1 transgenes were co-expressed in the best-responding spliceosensor line [*MHC*-Gal4>UAS-minigene:Luc, UAS-i(CTG)_480_, UAS-MBNL1] (Fig. 2B). Luciferase activity increased by 46.4% (INSR:Luc#6), 27.8% (cTNT:Luc#8) and 28.4% (TnnT3:Luc#7) following human MBNL1 expression compared to disease control expression [*MHC*-Gal4>UAS-minigene:Luc,UAS-i(CTG)_480_,UAS-GFP]. These results point to a dependence on human MBNL1 in the DM1 alternative splicing events used in the fly biological context, which, taken together with our previous observations, imply the conservation of the DM1 pathogenic pathway in *D. melanogaster* and support the use of these flies as DM1 spliceosensors.

### Characterization and validation of DM1 *INSR* spliceosensor line #6

**Fig. 3.**
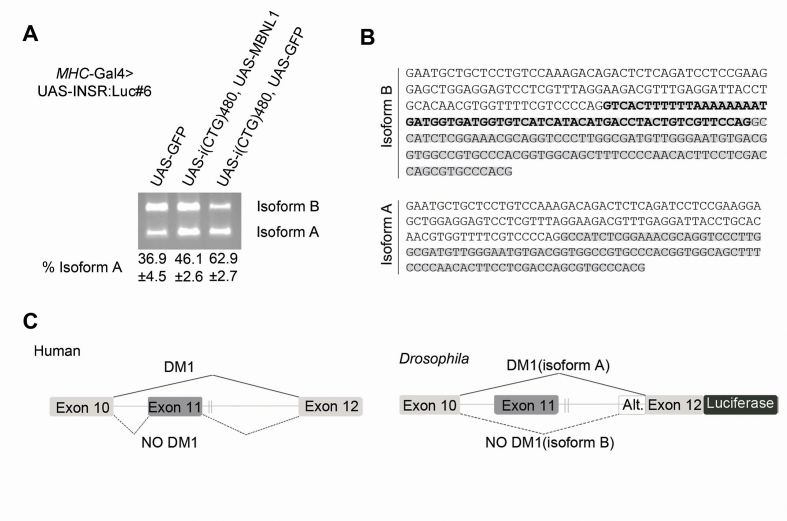
**Validation of *INSR* spliceosensor flies.** (A) RT-PCR results of *MHC*-Gal4>UAS-INSR:Luc#6 spliceosensor expression under different genetic backgrounds: in the presence of the CUG repeat RNA expression alone [UAS-i(CTG)_480_, UAS-GFP], simultaneously with the CUG repeat RNA and human MBNL1 [UAS-i(CTG)_480_, UAS-MBNL1], or in a no DM1 state (UAS-GFP). (A) Two PCR isoforms (isoform A and isoform B) were observed after amplification, but in different proportions for each genotype analyzed. Results are given as percentages (±s.d.) below the image. (B) Fly sequences from the two isoforms identified in A. Isoform B included exon 10 (unmarked), exon 12 (marked in gray) and an additional 62 nucleotides of intronic sequence immediately upstream from exon 12 (bold). Isoform A contained exon 10 (unmarked) and 12 (marked in gray). (C) Schematic of the *INSR* alternative splicing established in humans and on the transgene in flies. Isoform A is favored in the DM1 state (solid lines) and isoform B is favored in a no DM1 state (dashed lines).

From the best spliceosensor flies above described, the largest recovery in luminescence activity following MBNL1 expression and the smallest variability between replicas was detected in *INSR* spliceosensor line #6 (*MHC*-Gal4>UAS-INSR:Luc#6), here defined as the best option for large-scale chemical screening (Fig. 2A,B). To further characterize this line, we correlated the luciferase levels with the splicing isoforms being transcribed. For this purpose, we performed RT-PCR analysis on the same fly genotypes as for Fig. 2B, reflecting different disease states. As previously observed in humans (Santoro et al., 2013), RT-PCR analysis demonstrated two isoforms (A and B) for the *INSR* transgene (Fig. 3A). In the no DM1 state (cross with UAS-GFP, no repeat presence) the relative amount revealed lower levels of isoform A (36.9%). In contrast, in the presence of toxic CUG RNA [cross with UAS-i(CTG)_480_, UAS-GFP] isoform A represented 62.9% of the *INSR* expression. We also observed recovery to a no DM1 state after MBNL1 co-expression (the isoform A amount decreased to 46.1% after crossing with UAS-i(CTG)_480_, UAS-MBNL1) (Fig. 3A). Notably, the luciferase levels correlated very well with the relative amounts of the *INSR* splice isoforms detected. Further sequencing of PCR amplicons revealed a variation in the *INSR* splicing outcome from *Drosophila* samples (Fig. 3B,C). Nucleotide sequencing of isoform B (top band), predominant in the no DM1 state and which results in exon 11 inclusion in humans, found exon 11 skipping plus the inclusion of 62 nucleotides upstream of exon 12 in flies (Alt in Fig. 3C). This result suggested the use of a cryptic 3′ splicing site in the human *INSR* intronic sequence, perhaps because of the different accessibility to a human sequence of the alternative splicing machinery present in *Drosophila*. At the same time, no differences were detected in the splicing outcome (exon 11 skipping) between human and flies after the sequence analysis of isoform A (predominant in the DM1 state when muscleblind proteins are sequestered by CUG repeats). Taken together, *INSR* minigene splicing processing in *D. melanogaster* was strongly reliant on CUG repeat expression, was successfully rescued by human MBNL1 splicing factor and did not alter the luciferase reading frame from that in the initial design.

To establish the suitability of the DM1 *INSR* spliceosensor fly model chosen for pharmacological testing, we evaluated two small molecules previously described as being missplicing modifiers in DM1. Pentamidine moderately rescued skipping of the *INSR* exon 11 after minigene transfection in HeLa cells, but it showed *in vivo* toxicity in mice (Warf et al., 2009). Flies behaved similarly, with important mortality issues also detected. Pentamidine treatments limited to final very low concentrations produced no enhancement of luciferase expression (supplementary material Fig. S1A). Triciribine, a molecule capable of partially restoring a cell-based *CLCN1*-luciferase splicing system linked to DM1 (O’Leary et al., 2010), was also tested. Under our *in vivo INSR* spliceosensor assay conditions, triciribine treatment led to the recovery of luminescence levels (supplementary material Fig. S1B). These data supported the competence of the transgenic INSR:Luc#6 flies not only for the identification of active molecules, but also for the detection of potential deleterious effects, and the model provided a reliable spliceosensor screening tool for drug discovery.

### *Drosophila*-based platform set-up: high-throughput screening design

**Fig. 4.**
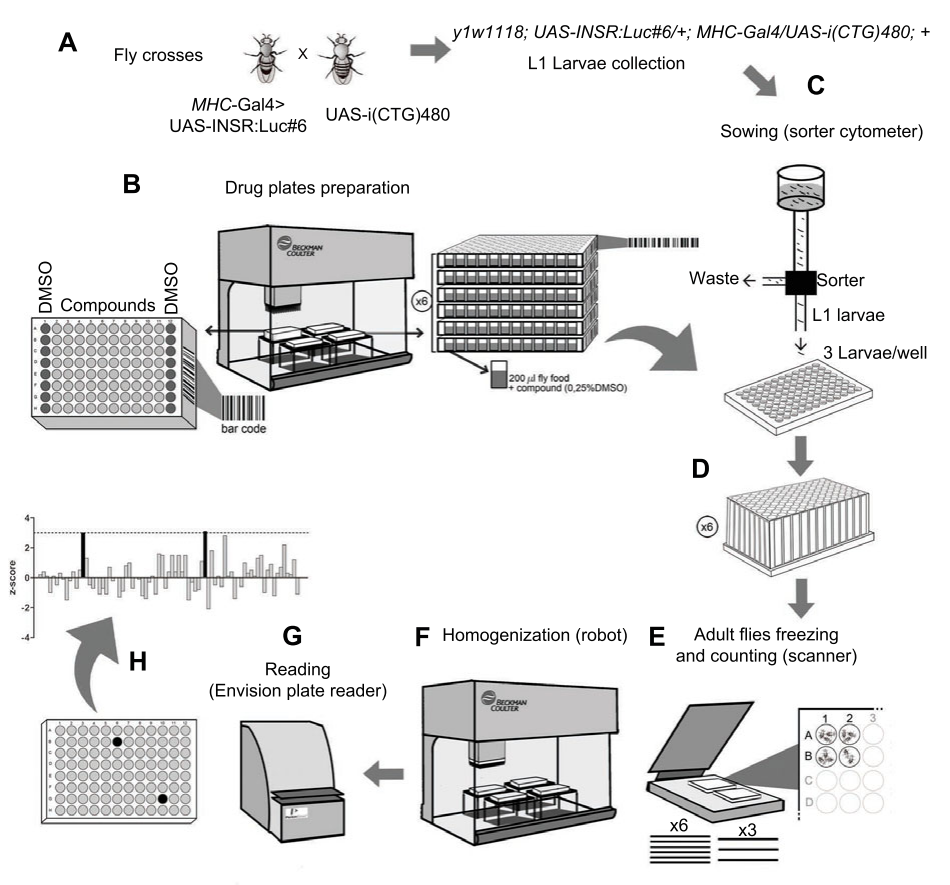
**Set up of the *Drosophila*-based HTS platform**. (A) A mass cross of *MHC*-Gal4>UAS-INSR:Luc#6 flies with UAS-i(CTG)_480_ flies was performed for the collection of first-instar larvae (L1) with the simultaneous expression of the toxic CUG repeat and the spliceosensor *INSR* transgene [*y*^1^ *w*^1118^; *UAS-INSR:Luc#6/+*; *MHC-Gal4/UAS-i(CTG)_480_*; +]. (B) Compounds and *Drosophila* food were dispensed in 96-well flat-bottomed plates using a liquid multidispenser robot. For optimal assay conditions, six plate replicates (x6) were performed. (C) Three L1 per well were plated using a sorter and each plate was covered with an inverted 96-well plate with deep wells. (D) Flies were allowed to grow for 14 days at 25°C, and at this point plates were inverted and frozen. (E) The number of flies per well in each replicate was counted using a scanner. (F) Flies were homogenized using the robot to ensure equal homogenization in all wells. (G) Lysate luminescence levels were measured with an Envision plate reader and normalized by dividing by the number of flies in each well. (H) These data were used to calculate the *z*-score for each compound in the plate. All plates used in the screening were identified with barcodes and checkpoints were inserted prior to each instrument usage.

At this point, we looked to combine the advantages of a screening assay with the use of our *in vivo* spliceosensor approach. Most pharmacological screens described using *D. melanogaster* have been manually performed (Pandey and Nichols, 2011). To accomplish high-throughput *in vivo* drug testing, the method required both miniaturization and automation steps (Fig. 4; supplementary material Fig. S2). Several parameters concerning compound dispersal, fly development competition issues and assay format, were tested and optimized. A screening format was established using 96-well plates, each one containing 200 μl of fly culture medium with 5 μl of the test compound diluted to a non-toxic final concentration of 0.25% in DMSO (Fig. 4B; supplementary material Fig. S2B). The most favorable condition for fly development was with three first-instar larvae (L1) seeded per well (supplementary material Fig. S2A). The 96-well plate miniaturized approach combined with the reporter-based readout system allowed for automation of the screening process by using (i) a liquid-handling robot station (Biomek NXP, Beckman Coulter, Brea, CA, USA) to distribute *Drosophila* food and chemical compounds, and to homogenize adult flies (Fig. 4B,C,F), (ii) a COPAS sorter cytometer (Union Biometrica, Holliston, MA, USA) used for automated dispensing of L1 larvae (Fig. 4C) resulting from a mass fly cross (Fig. 4A), (iii) a digital scanner (Epson Expression 10,000 XL, Suwa, Japan) for image analysis and quantification of the final number of adult flies per well (Fig. 4E), (iv) a plate reader (Envision 2104, Perkin Elmer, Waltham, MA, USA) for accurate luminescence quantification from fly homogenates (Fig. 4F), and (v) barcodes for all plates, as well as barcode checkpoints introduced prior to each robot usage (Fig. 4B). Robotic implementation of the whole screening process increased the throughput per day and reduced potential human errors. The final readout time-point was adjusted to 14 days (fly growing conditions at 25°C) from the sorting day, when most of the adult flies had already emerged from the pupae. At this point, flies were routinely frozen, counted with the digital scanner, and homogenized with the liquid-handler robot (Fig. 4D–F). Finally, luminescence levels were measured with the Envision plate reader (Fig. 4G). Automated sorting did not distinguish sex, because the differences in luciferase levels were not high enough to give false positives. The ratio between luminescence units (relative light units, RLU) and the number of flies in each well was used to calculate the percentage of recovery for each tested compound, indicating the potential biological activity on the established *in vivo* DM1 spliceosensor assay (Fig. 4H).

Finally, the *in vivo* screening method involved the automated reading of a whole 96-well plate containing untreated (only DMSO in the nutritional medium) flies (three per well) that expressed the *INSR* spliceosensor, but not the toxic CUG repeat (*MHC*-Gal4> UAS-INSR:Luc#6). These flies were used as positive controls to calibrate the upper luciferase levels (high-grade entry for gene-reporter activity) and efficiently calculate the *Z*-factor value for each independent assay batch (Zhang et al., 1999). Consecutively, 96-well plates containing testing compounds were filled with flies that simultaneously expressed the *INSR* spliceosensor and the toxic CUG repeat [*MHC*-Gal4>UAS-INSR:Luc#6, UAS-i(CTG)_480_]. Each plate layout included 16 positions containing only 0.25% DMSO, configured as internal negative control wells in order to measure gene reporter levels in the absence of any compound (Fig. 4B, low-grade entry for minigene-reporter activity), and 80 wells where compounds were tested separately. This configuration allowed the establishment of a screening window for the reliable assessment of the ability of a compound to lead to the recovery of luciferase expression. To satisfy statistical validation requirements, we tested six identical replicates for each of the 80 compounds (*Z*-factors between 0 and 1) (Fig. 4B; supplementary material Fig. S3A). On a daily basis, the *in vivo* assay involved reading nearly 2000 data points (three distinct 80-compound panels), reaching screening capabilities for the reliable evaluation of 720–1200 compound batches weekly.

### Screening campaign results by using the DM1 *INSR* spliceosensor assay

**Fig. 5.**
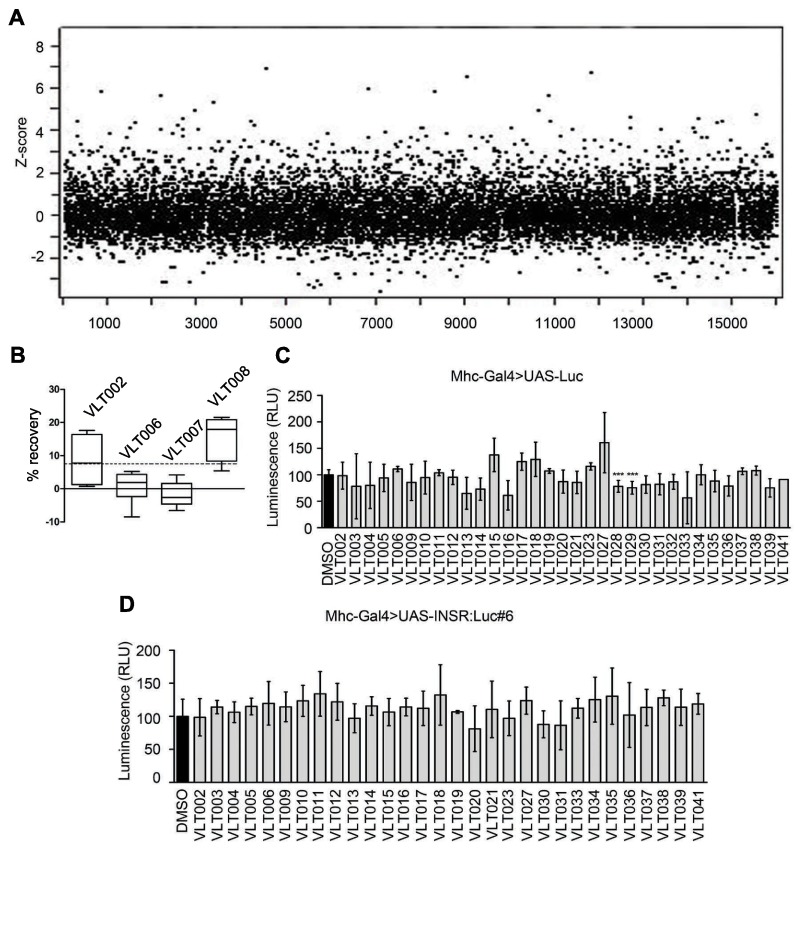
**Primary screening results and hit confirmation steps.** (A) Scatter plot of *z*-scores obtained for the 16,063 compounds originally screened, showing several compounds able to reach *z*-scores ≥3. (B) Representative example of results achieved from cherry-picked VLT primary hits in the confirmatory activity study, graphed as boxes representing percentiles (minimum to maximum) with the average marked as a straight line. The initial positive splicing modulator activity was confirmed for VLT002 and VLT008. All the individual flies tested displayed an enhancement of luciferase levels, with an average increase greater than 7.5% [dashed line, statistical threshold after Student’s *t*-test (*n*>4 flies)]. (C) Secondary assay based on the quantification of luciferase levels after treatment with the VLT hits in *MHC*-Gal4>UAS-Luc flies. The RLU normalized to DMSO treatment is plotted. Two compounds (VLT028 and VLT029) significantly changed luminescence levels and were discarded from further validation steps (*n*>4 flies). ***P<0.001 (Student’s *t*-test). (D) Secondary assay based on quantification of luciferase levels after VLT hit treatments in *MHC*-Gal4>UAS-INSR:Luc#6 flies, expressing the spliceosensor transgene independently of the CTG repeat induction. The RLU normalized to DMSO treatment is plotted. No compound significantly changed spliceosensor luminescence levels (*n*>4 flies).

Having established the robustness and sensitivity of the *Drosophila* DM1 spliceosensor primary screening assay, we performed an initial screening evaluation on eight plates from the Prestwick chemical library (640 compounds). Positive *Z*-factor values assured the quality of the assay (Zhang et al., 1999) (supplementary material Fig. S3A). Results were analyzed as *z*-score values (Kreyszig, 1979) (supplementary material Fig. S3B) after checking that the recovery values were normally distributed (*W*=0.97 in a Shapiro-Wilk *W* test) (supplementary material Fig. S3C). The assay was able to detect several molecules displaying more than a threefold increase in luminescence levels (*z*-score values≥3) (supplementary material Fig. S3B). This restrictive value was defined as the threshold for identification of the screening hits. Based on the performance of the platform after the initial 640 compounds, we followed up with a large-scale primary screen using the parameters optimized herein. In total, 16,063 different chemical entities were screened for their ability to rescue *INSR* CUG-induced missplicing, read as a significant luminescence increase in DM1 *INSR* spliceosensor flies. All individual batches with a positive *Z*-factor were considered for further analysis. After checking that the recovery values were normally distributed [D=0.07 in a Kolmogorov–Smirnov– Lilliefors (KSL) test], the *z*-score value was calculated for each compound (Fig. 5A). A total of 126 compounds were identified as screening hits (*z*-score value≥3), that resulted in a hit rate of 0.78% from this primary screen (defined as VLT hits).

### Hit validation by secondary screenings

Luciferase levels were normalized by dividing by the number of flies per well. This method reduces costs and variability, but it can also lead to identification of false positives. Furthermore, muscle or total body mass could nonspecifically increase luciferase levels after compound treatment and, therefore, be identified as positives. In order to corroborate the level of compound activity and to discard potential false positives, hits were routinely and rapidly tested on successive assays. First, they were retested for activity by using the same DM1 *INSR* spliceosensor assay, but this time assessing luminescence levels from individual flies (a minimum of four replicates). A consistent increase in reporter levels from most of the flies tested individually, combined with an average enhancement of luciferase activity above 7.5% [confirmed as significant after applying the Student’s *t*-test (*P*>0.05)] was set as the assay threshold for hit activity validation (Fig. 5B). Up to 32 VLT hits showed enhanced luciferase levels that ranged from 7.5% to 20% increases. Second, we filtered out hits able to directly modulate the transcription of the UAS-Gal4 system or to influence the minigene reporter activity. Thus, flies expressing only luciferase (*MHC*-Gal4>UAS-Luc) or only the minigene (*MHC*-Gal4>UAS-INSR:Luc#6) were treated with the 32 VLT hit panel. Two molecules (VLT028 and VLT029) significantly changed luciferase readings independently of the presence of the *INSR* minigene (Fig. 5C) and were therefore excluded from further validation. None of the compounds changed the *INSR* splicing in the absence of CUG repeat RNA (Fig. 5D). Consequently, 30 of the primary 126 identifications were considered to be confirmed VLT hits (0.19% final hit rate). Examples of some molecules identified in our *in vivo* screening, along with their structures, are shown in the supplementary material Fig. S4.

### Evaluation of the effect of the hit compounds on well-known DM1 hallmarks

**Fig. 6.**
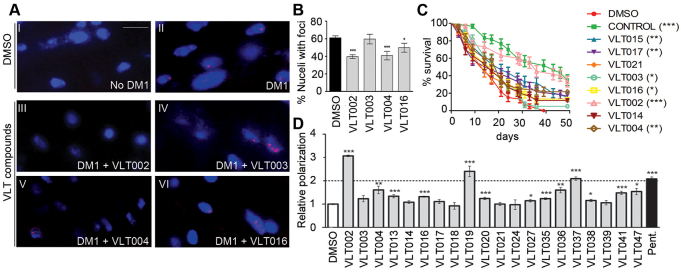
**Hit evaluation on well-known DM1 hallmarks.** (A) Thorax transversal sections of (I) no DM1 (*MHC*-Gal4>UAS-GFP) and (II) DM1 [*MHC*-Gal4 >UAS-i(CTG)_480_] flies treated with DMSO, and (III–VI) DM1 [*MHC*-Gal4>UAS-i(CTG)_480_] flies treated with VLT confirmed hits. CUG nuclear aggregations (foci) were consistently detected by FISH (red dots) in ~60% of the nuclei (stained in blue) in DM1 flies (II), but were not present in controls (I). Treatment with VLT003 hit (IV) did not cause a decrease in the number of foci. However, VLT002 (III), VLT004 (V) and VLT016 (VI) treatments visually reduced the number of foci. Scale bar: 10 μm. (B) Graphic representation of the percentage of nuclei with foci after VLT hit treatments.. Error bars represent s.e.m. (*n*>3 flies per treatment and at least 50 cells counted from each one). (C) Lifespan of DM1 flies treated with different hits. MHC-Gal4>UAS-i(CTG)_480_ flies treated with DMSO, the indicated VLT compound or control (*yw*) flies treated with DMSO. The percentage of MHC-Gal4>UAS-i(CTG)_480_ flies treated with only DMSO that survived was significantly lower than wild-type flies (*yw*). Several compounds were able to increase the lifespan of these DM1 flies, and one compound totally rescued this phenotype achieving wild-type survival rates. (D) A fluorescence polarization assay was used to establish the ability of confirmed hit compounds to bind a disease-length CUG RNA (CUG_23_-FAM RNA probe). Pentamidine was used as the positive control for the fluorescence polarization assay (black bar). The same or higher RNA polarization changes observed with pentamidine (twofold) compared to the negative control (DMSO) (white bar) was established as the positive binding threshold of the compounds tested. Three compounds (VLT002, VLT019 and VLT037) were identified as being able to bind with toxic RNA. Error bars represent s.e.m. (*n*=4 replicates). *P<0.05, **P<0.01, ***P<0.001 (Student’s *t*-test).

The formation of ribonuclear foci is a well-established DM1 disease hallmark in cells from DM1 patients, and has also been reproduced in the UAS-i(CTG)_480_ fly model used herein (García-López et al., 2008). It therefore can be used to screen and assess for potentially useful therapeutic compounds (Ketley et al., 2014). To assess whether some VLT hits could reduce foci numbers, we performed a quantification of the number of foci in *Drosophila* by using fluorescence *in situ* hybridization (FISH). We identified several compounds that significantly reduced the number of anomalous aggregates in muscle cells (Fig. 6A,B). To further validate the biological activity of the identified hits, some compounds were screened for their ability to ameliorate the short-lifespan phenotype of DM1 flies described previously (García-López et al., 2008). As shown in Fig. 6C, most of the hits analyzed were able to increase the lifespan of DM1 flies at different levels of significance [VLT002 (*P*<0.0001), VLT003 (*P*<0.005), VLT004 (*P*<0.005), VLT015 (*P*<0.0001), VLT016 (*P*<0.05) and VLT017 (*P*<0.001)] suggesting a positive response on functional disease signs. It is interesting to note that the proportion of VLT002-treated flies that survived was comparable to that of wild-type flies (no statistical differences between both curves), indicating that there is a total rescue of the lifespan of DM1 flies owing to the VLT002 treatment.

In parallel, we also evaluated the ability of VLT hits to target the toxic CUG-RNA, a mechanism of action linked to the reduction of DM1 phenotypes in disease models (Childs-Disney et al., 2012; Wheeler et al., 2012; García-López et al., 2011; Parkesh et al., 2012). Using a fluorescence polarization spectroscopy assay, previously used for studying binding of small molecules to the CUG-RNA hairpin structure (García-López et al., 2011), we identified some hits that exhibited this interesting aptitude (Fig. 6D). Taken together, these results confirm the ability of the screening method developed, based on a spliceosensor reporter system, to reliably identify novel molecules with promising therapeutic features for the treatment of DM1. These VLT hits are attractive candidates for translational research projects on DM1 disease.

## DISCUSSION

Manipulation of splicing by different means is considered a promising therapeutic approach to human disorders (Havens et al., 2013; Singh and Cooper, 2012), such as for DM1 where target-based identification of active antisense oligonucleotides and rational design of small molecules are actively pursued strategies (Wheeler et al., 2009; Childs-Disney et al., 2013). A different strategy is the development of high-throughput screening assays using cell culture systems to search for chemical modulators of specific missplicing events (Havens et al., 2013), a technique hardly tested for DM1 (O’Leary et al., 2010; Oana et al., 2013). Focusing on improving the often quite poor quality of hits resulting from traditional *in vitro* brute-force pharmacological screenings, the present work has established a novel *in vivo* screening methodology based on an automated platform for large-scale and cost-effective *Drosophila*-based evaluation of small molecules, pioneering the exploitation of a disease-linked alternative splicing reporter system. The method, initially devised for DM1 and potentially expandable to additional missplicing disorders, allows for the fast and reliable identification of splicing modulators after the successful validation of the first transgenic spliceosensor flies.

For modeling purposes, we took advantage of the competence of *D. melanogaster* both to allow a stable integration of human DNA in its genome and to mirror disease phenotypes (Pandey and Nichols, 2011; García-López et al., 2008; Xu et al., 2013; Demontis et al., 2013; Piccirillo et al., 2014). The ‘humanized’ spliceosensor flies accurately reproduced the alternative splicing deregulation described in DM1 patients in the presence of the disease mutation (Udd and Krahe, 2012; Ranum and Cooper, 2006). Simultaneously, we used the latest technology applied to small organisms to achieve *in vivo* HTS capabilities (Giacomotto and Ségalat, 2010). At this point, we determined the standards for valid screening parameters (positive *Z*-factor and a *z*-score≥3) on the use of the spliceosensor flies, thus allowing the assessment of chemical entities in a large-scale format. Importantly, some of the confirmed hits identified by positive modulation of the DM1 spliceosensor system were confirmed as molecules with positive roles in relevant independent DM1 assays, and for this reason they are currently the subject of further evaluation in additional DM1 systems.

At this point, the evaluation of treatments that simultaneously combine use of compounds with different anti-DM1 features or chemical properties is an experimental strategy in need of exploration in order to improve the potential anti-DM1 response. These confirmatory data suggest that our method is able to identify high-quality hits from screened compounds, one of the advantages anticipated from the use of whole animals in screening (Pandey and Nichols, 2011; Giacomotto and Ségalat, 2010).

The reliable use of a sensitive reporter-based system in an *in vivo* situation established a novel and interesting option for pharmacological evaluation in addition to previously established *Drosophila*-specific phenotypic outputs, such as behavioral assays, or assays of lethality or eye roughness, which are commonly limited to low- and medium-throughput assays, are difficult to miniaturize or automate and show higher heterogeneity in their final measurements (Pandey and Nichols, 2011; Giacomotto and Ségalat, 2010). Furthermore, the use of the spliceosensor flies offers the possibility of establishing distinct types of mechanistic outputs from the compounds identified. The screening herein described involved a splicing-phenotype-based approach, as induced by the expression of CUG repeats, without connecting the hit evaluation to a specific mechanism of action. That said, a screening approach that is closer to a target-based approach is also possible by using the spliceosensor flies alone and looking for direct modulators of the splicing event. Given that our approach entails a high-throughput (HTS) format, the *in vivo* screening capabilities exhibited are very promising. Most pharmacological screens described in *Drosophila* are on the order of 500 to 1000 molecules tested per month (~15 to 30 daily) (Pandey and Nichols, 2011). Our approach is around a 10-fold increase in the *in vivo* throughput because we were able to test 240 compounds daily.

A limitation on the development of *in vivo* screening methods is the unfeasibility of traditional brute-force traditional methods that usually involve mass 384-, 1536- or 3456-well plate formats. In contrast, *Drosophila*-based screening methods offer the ability to test compound activity directly in a living animal with the simultaneous evaluation of toxicity and drug-like properties. Moreover, and of significance, the use of flies allows for an accurate control of the expression system, as demonstrated here by targeting the DM1 transgenes only in somatic muscles, a key tissue in DM1 progression.

One potential drawback to the use of *Drosophila* is linked to the extent of genome and pathway conservation, although this organism does display a high degree of conservation in genes, structures and functional processes characterized in vertebrate skeletal muscle (Tixier et al., 2010). This makes *Drosophila* particularly well-suited to modeling and studying muscular disorders (García-López et al., 2008; Lloyd and Taylor, 2010; Mosqueira et al., 2010; Timmerman and Sanyal, 2012). Regarding alternative splicing, although important functional conservation occurs between *Drosophila* and mammals (Venables et al., 2012), dissimilarities have also been described (Mount et al., 1992; Irion, 2012). Results obtained here from the DM1 spliceosensor flies suggested, in this specific condition, that there were only slight alternative splicing machinery differences, hence, still allowing for robust disease mirroring. Noticeably, transgenes were strongly reliant on CUG repeat expression and successfully rescued by co-expression of human MBNL1. The best corroboration of the effectiveness of the screening method was the confirmation of positive activity on splicing-independent key DM1 features for some compounds, such as the ability to bind to the toxic CUG-RNA or to reduce the number of foci aggregates. Taken together, this suggests that the *in vivo* drug discovery approach demonstrated here could significantly reduce post-screening costs for identifying quality leads from the initial candidate pool.

Success in the identification of novel valid compounds for the potential development of a DM1 treatment suggests that the method developed could be adapted to any particular type of alternative splicing deregulation (exon skipping, intron retention and exon extension, among others) linked to human disease, such as in myotonic dystrophy type 2 (DM2) (Udd et al., 2011), progeria (Beard et al., 2008), Alzheimer’s disease (Crowe et al., 2009) or cancer (Clower et al., 2010), where missplicing events are already well-described and for which key disease aspects are conserved in *D. melanogaster* (Pandey and Nichols, 2011). We foresee its extended use in genetic screens focused on better understanding the mechanisms of splicing misregulation in human disease. The versatile use of reporter-based platforms in whole organisms, where it is at the moment still very limited to cell culture (Younis et al., 2010; Arslan et al., 2013; Zheng et al., 2013; O’Leary et al., 2010), should serve to rapidly expand the kind of *in vivo* HTS screens for which *D. melanogaster* can be widely used.

## MATERIALS AND METHODS

### *Drosophila* stocks

UAS-MBNL1 (García-Casado et al., 2002) and UAS-i(CTG)_480_ (García-López et al., 2008) flies were previously described and available at our laboratory. *MHC*-Gal4 flies were a kind gift from Eric Olson (University of Texas, Southwestern Medical Center, TX). UAS-GFP flies were from Bloomington *Drosophila* Stock Center. *Drosophila* stocks were grown at 25°C in standard fly food (the recipe can be found on the Bloomington website, http://flystocks.bio.indiana.edu).

### Generation and management of DM1 spliceosensor flies

UAS-Luc stable transformant flies were generated by cloning the firefly luciferase open reading frame (ORF) from the pGL3-Enhanced vector (Promega, Fitchburg, WI) into the *Xho*I and *Xba*I sites of the pUAST vector (Brand and Perrimon, 1993) and transgene microinjection into *y*^1^ *w*^1118^ embryos (BestGene, Chino Hills, CA). For spliceosensor fly generation, UAS-INSR:Luc, UAS-cTNT:Luc and UAS-TnnT3:Luc transgenes were generated by site-directed PCR mutagenesis of the human *INSR* minigene (a gift from Dr Nicholas Webster, University of California, San Diego, CA), the human *cTNT* minigene (a gift from Dr Tomas Cooper, Baylor College of Medicine, Houston, TX), the mouse *TnnT3* minigene (a gift from Dr Maurice Swanson, University of Florida, College of Medicine, FL) and pGL3 (Promega, Fitchburg, WI). Minigene modifications were introduced into the 5′ primer as follows: a *Eco*RI restriction site, a Kozak sequence (ACCATGG) to increase protein translation, a translation initiation codon ATG and, only in the *INSR* minigene, two nucleotides (CC) to correct the luciferase ORF. Similarly, the *Xho*I restriction site adapter was introduced into the 3′ primer. Luciferase modifications were introduced into the 5′ primer (*Xho*I restriction site and deletion of its translation initiation codon ATG) and the 3′ primer (*Kpn*I restriction site) (Fig. 1A; supplementary material Table S2). Modified luciferase was cloned into the *Drosophila* pUAST vector (pUAST-Luc) after sub-cloning into the pJET vector (Thermo Scientific, Waltham, MA). Modified *INSR*, *cTNT* or *TnnT3* minigenes were cloned into the pUAST-Luc after being sub-cloned into the pJET vector. Transgene microinjections were performed as described above.

Transgene genomic location was assessed by inverse PCR accordingly to the method described at http://www.fruitfly.org/about/methods/inverse.pcr.html (Berkeley *Drosophila* Genome Project). Spliceosensor flies will be available for non-commercial applications and sent to academic and nonprofit organizations upon request. Mass first-instar (L1) larvae [*yw*; *UAS-INSR:Luc#6/+*; *MHC-Gal4/UAS-i(CTG)_480_*] harvesting, needed for the spliceosensor screening assay, was performed in cages containing egg collection plates at the bottom. Eggs were allowed to develop on these collection plates (1.7% agar, 2.6% ethanol and 0.86% acetic acid in water) until the L1 stage and were then collected with water washes prior to their automated plating.

### Reverse transcription-PCR analysis

Total RNA was extracted from ~50 adult flies with Tri-Reagent (Sigma, St Louis, MO) following the manufacturer’s instructions. Contaminating DNA was degraded by RNase-free DNase I (Thermo Scientific, Waltham, MA). Reverse transcription was performed with Superscript II polymerase (Invitrogen, Carlsbad, CA) following the manufacturer’s guidelines. Finally, Gotaq polymerase (Promega, Fitchburg, WI) was used for PCR amplification with primer sequences and conditions as described in supplementary material Table S2.

### *In vivo* high-throughput screening

#### Screening parameters

For drug administration, *Drosophila* larvae were fed by dispensing the compound directly mixed with the nutritive medium. Three L1 larvae were chosen as the best option for assay progression without compromising adult viability and HTS screening conditions (supplementary material Fig. S2A). Dimethyl sulfoxide (DMSO), the most frequently employed solvent for chemical libraries, was used at 0.25% to prevent toxicity (Nazir et al., 2003) (supplementary material Fig. S2B).

#### Assay configuration of the 96-well plates

First, 5 μl of any of the chemical compounds was added to flat-bottomed 96-well plates (Daslab, Barcelona, Spain) and mixed with 200 μl of standard fly food by using a Biomek NXP liquid-handling robot station (Beckman Coulter, Brea, CA). Secondly, L1 larvae [*yw*; *UAS-INSR:Luc#6/+*; *MHC-Gal4/UAS-i(CTG)_480_*] were plated in each well using a mid-size flow cell cytometer (COPAS select, Union Biometrica, Holliston, MA). Finally, each plate was sealed by placing a micro-perforated 96-well plate with deep wells (ABgene, Waltham, MA) in an upside-down position with a punched foam placed between both plates to avoid larvae from passing from one well to another. Six replicates were assayed from each screening plate. All plates, including those later used for the luciferase read-out (see below), were labeled with barcodes for correct sample identification throughout the entire screening process. Flies were allowed to develop in a compound containing food at 25°C for 14 days. At this point, flies reached the adult stage and were frozen at −20°C, prior to quantifying their minigene luciferase levels.

#### Luciferase read-out

For primary screening, frozen flies were manually transferred to a new flat-bottomed 96-well plate (Daslab, Barcelona, Spain) by inverting the deep-well plate over this new plate. At this point, replicates from the initial six were combined to create three replicates for the final read-out. The number of flies per well was counted using the Epson Expression 10000 XL scanner. After quantification, 150 μl of 1× reporter lysis buffer (Promega, Fitchburg, WI) was added to each well using the Biomeck NXP liquid multidispenser robot (Beckman Coulter, Brea, CA), which was also used to homogenize flies. Then, 50 μl of the homogenate was transferred to a new white 96-well plate (Sterilin, South Wales, UK). Lysate luminescence was measured with the Envision plate reader (Perkin Elmer, Waltham, MA) after dispensing 15 μl of Luciferase Assay Reagent (Promega, Fitchburg, WI) with the Envision injector. The final data collected for each well was its luminescence (RLU, relative light units) normalized to the number of flies counted in the well. For all other luciferase measurements, individual flies were manually placed in each well prior to homogenization; therefore, the final data analyzed were equal to their luminescence.

#### Screening statistics

For primary screening, the *Z*-factor of each plate was calculated using the formula previously described (Zhang et al., 1999): *Z*=1–[(3 s.d. sample + 3 s.d. control)/|mean sample – mean control|]. *MHC*-Gal4>UAS-INSR:Luc#6 flies fed with DMSO were the ‘sample’ and *MHC*-Gal4>UAS-INSR:Luc#6, UAS-i(CTG)_480_ flies fed with DMSO were the ‘controls’. Robustness of the test was verified for each plate with the identification of a positive *Z*-factor (otherwise, the plate was repeated). For compound ranking calculations, first, the percentage of recovery compared to DMSO-treated *MHC*-Gal4>UAS–INSR:Luc#6, UAS-i(CTG)_480_ flies was calculated for each compound. The normal distribution for all percentages of recovery was calculated using JMP 7 (statistical analysis software, Cary, NC). Then, the *z*-score was calculated for each one using the following formula (Kreyszig, 1979): *z*-score = (*X*_i_–*X*)/*s*, where ‘*X*_i_’ is the percentage of recovery of the compound tested, and ‘*X*’ and ‘*s*’ are the mean and standard deviation of all the recovery percentages on the plate. Positives were prioritized for 3 s.d. from the mean of the normalized luciferase activity of each plate (i.e. *z*-score of 3 or higher). For all other luminescence measures, including validation of second-stage hits, percentages of recovery were analyzed using a non-paired Student’s *t*-test (*P*<0.05). The Student’s *t*-test was also used to compare survival rates and number of cells with foci.

### Fluorescence *in situ* hybridization

*MHC*-Gal4>UAS-i(CTG)_480_ L1 larvae were plated under screening conditions onto plates containing either compound or DMSO. Adult flies (14 days after plate sorting) were used for the detection of ribonuclear foci. As a negative control of the technique, *MHC*-Gal4>UAS-GFP flies (DMSO) were also analyzed. Thoraces were embedded in Optimal Cutting Temperature reagent (OCT, Tissue-Tek) and transversal sections (12 μm) were taken with a Leica CM 1510S cryomicrotome. Sections were collected on super-frost slides (Thermo Scientific, Waltham, MA) and washed three times for 5 minutes with PBS-DEPC. Background reduction was accomplished with a 10-minute wash in acetylation buffer (0.1 M triethanolamine, 0.25% acetic anhydride), followed by another three DEPC-treated PBS washes of 5 minutes each. Prehybridization was performed for 30 minutes in hybridization buffer (50% deionized formamide, 0.3 M NaCl, 20 mM Tris-HCl, pH 8, 5 mM EDTA, 1× Denhardt’s solution, 10% dextran sulfate and 500 μg/ml herring sperm DNA) at room temperature. The hybridization probe (Cy3-CAGCAGCA-GCAGCAGCAGCA-Cy3) (Sigma, St Louis, MI) was denatured for 5 minutes at 65°C and snap-chilled on ice prior to its addition to new hybridization buffer (1:100), which replaced the buffer used in the prehybridization. Hybridization was performed overnight in a dark chamber at 37°C under humid conditions. After several washes (two of 15 minutes with 2× SSC at 32°C, two of 15 minutes with 0.5× SSC at 32°C and three of 5 minutes with PBS-DEPC at room temperature), sections were mounted with Vectashield mounting medium with DAPI (Vector Laboratories, Burlingame, USA). At least five 40× images of different focal planes along the *z*-axis were taken in a Leica DM2500 microscope for DAPI (UV channel) and Cy3 (green channel). The *z*-planes were stacked using Photoshop and the number of nuclei with foci counted with ImageJ software. At least 50 cells from each individual were counted and at least three individuals were analyzed for each compound. The percentage of cells with foci was compared between that for DMSO or for compound-treated flies using a non-paired Student’s *t*-test (Graph pad, Prism).

### Lifespan assay

A total of 15 newly hatched *MHC-Gal4>UAS-i(CTG)_480_* males were placed in vials containing compound or DMSO dissolved in standard *Drosophila* medium. As a positive control, 15 *yw* males were also placed in vials with DMSO dissolved in standard *Drosophila* medium. Four replicates were performed for each compound, giving a total number of 60 flies analyzed per compound. Flies were transferred every 2–3 days into fresh vials, when the number of survivor flies was scored. Kaplan–Meier survival curves were generated by plotting the number of survival flies as a function of time in days. Curves comparison was performed using the Kaplan–Meier test (Graph pad, Prism)

### Fluorescence polarization assay

FAM-CUG_23_ (Metabion, Martinsried, Germany) was annealed at 70°C for 10 minutes and allowed to cool slowly on the bench top. After cooling to room temperature, 6 nM of FAM-CUG_23_ was incubated with compound (1 mM) or 1% DMSO (free RNA controls) in binding buffer (25 mM Tris-HCl pH 7.5, 100 mM NaCl, 5 mM MgCl_2_, 50 μM ZnCl_2_, 10% glycerol and 0.05% Tween 20) on ice (20 minutes) in the dark. Pentamidine (P0547-Sigma, St Louis, MO), a nucleic-acid-binding small compound (Warf et al., 2009) was added as a positive control for each experiment. FAM-CUG_23_ polarization was measured in the Envision plate reader (Envision 2104, Perkin Elmer, Waltham, MA) using FP480 (excitation) and FP535 (emission) filters. Millipolarization (mP) values were calculated for each compound [mP=1000×(S–*G*×P)/(S+*G*×P)], where S and P were the fluorescence counts rated on the planes parallel (S) or perpendicular (P) to the excitation filter, and *G* (grating factor) was an instrument-dependent factor. Results were normalized to that of the negative (DMSO) control, and those compounds showing the same or having superior increases in polarization values relative to those for pentamidine polarization were scored as positives for CUG-RNA hairpin binding.

## Supplementary Material

Supplementary Material
