# Supplementary material for: Development of a Drosophila melanogaster spliceosensor system for in vivo high-throughput screening in myotonic dystrophy type 1
Source: Dis Model Mech. 2014 Sep 19;7(11):1297–306. doi: 10.1242/dmm.016592 (PMC4213733; doi:10.1242/dmm.016592)
Supplement: Supplementary Material [file supp_7_11_1297__index.html]

Development of a Drosophila melanogaster spliceosensor system for in vivo high-throughput screening in myotonic dystrophy type 1 — Supplementary Material 

# Development of a *Drosophila melanogaster* spliceosensor system for *in vivo* high-throughput screening in myotonic dystrophy type 1

## DMM016592 Supplementary Material

**Files in this Data Supplement:**

- **Supplementary Material**
