# Supplementary material for: Development of a Drosophila melanogaster spliceosensor system for in vivo high-throughput screening in myotonic dystrophy type 1
Source: Dis Model Mech. 2014 Sep 19;7(11):1297–306. doi: 10.1242/dmm.016592 (PMC4213733; doi:10.1242/dmm.016592)
Supplement: Supplementary Material [file supp_7.11.1297_DMM016592.pdf]

SUPPLEMENTARY FIGURE 1

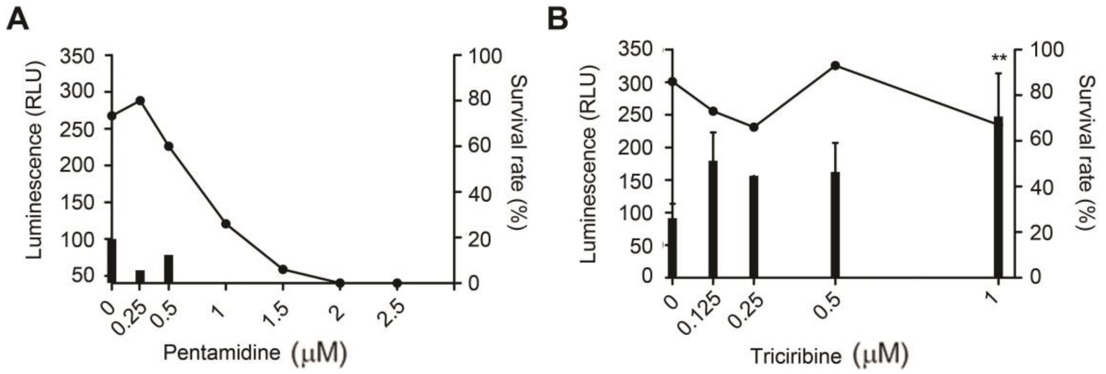

Supplementary Figure 1. Evaluation of the applicability of the *in vivo* DM1 INSR *spliceosensor* assay for pharmacological screening. (**a**) A dose-response for pentamidine was evaluated on *MHC*-Gal4>UAS-INSR:Luc#6, UAS-i(CTG)$_{480}$ *spliceosensor* flies. Survival rate (right axis) displayed strong toxicity at concentrations higher than 0.5 μM. Pentamidine treatment did not significantly change *spliceosensor* luciferase levels (left axis) at sub-toxic concentrations. (**b**) Triciribine was also evaluated in same flies and assay format. Luciferase levels displayed quantifiable increment in concentrations tested (left axis) and no toxicity issues were detected (right axis). **p-value < 0.005 obtained by an unpaired t-test.

SUPPLEMENTARY FIGURE 2

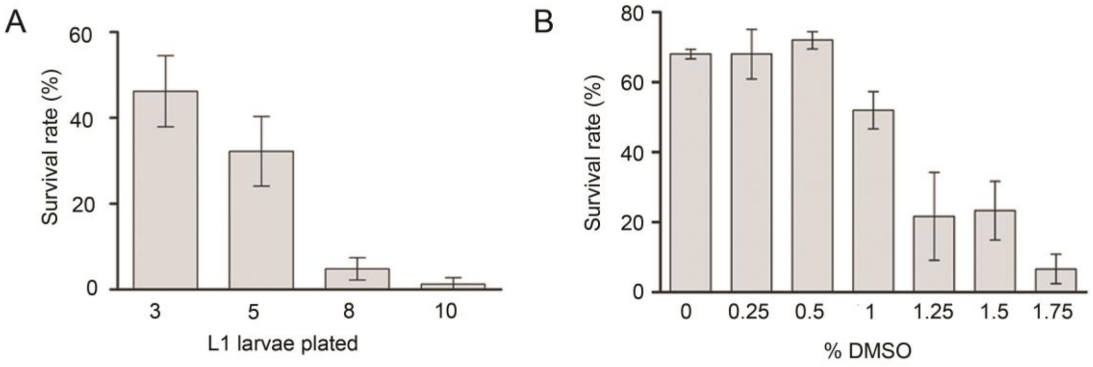

Supplementary Figure 2. Adjustment of *Drosophila* culture conditions to a miniaturized screening format. (**a**) Optimization of the number of L1 larvae developed per well. Survival rate of *MHC*-Gal4>UAS-INSR:Luc#6, UAS-i(CTG)$_{480}$ adults flies was assessed for three, five, eight and ten larvae per well in a 96-well plate format, showing best survival rate when three larvae per well were plated. (**b**) Assessment of adult fly toxicity levels associated to DMSO final amount. *MHC*-Gal4>UAS-INSR:Luc#6, UAS-i(CTG)$_{480}$ L1 larvae were seeded in nutritive media with increasing DMSO concentration (0 to 1.5 %). Concentrations lower than 0.5% DMSO do not significantly decreased fly surveillance.

SUPPLEMENTARY FIGURE 3

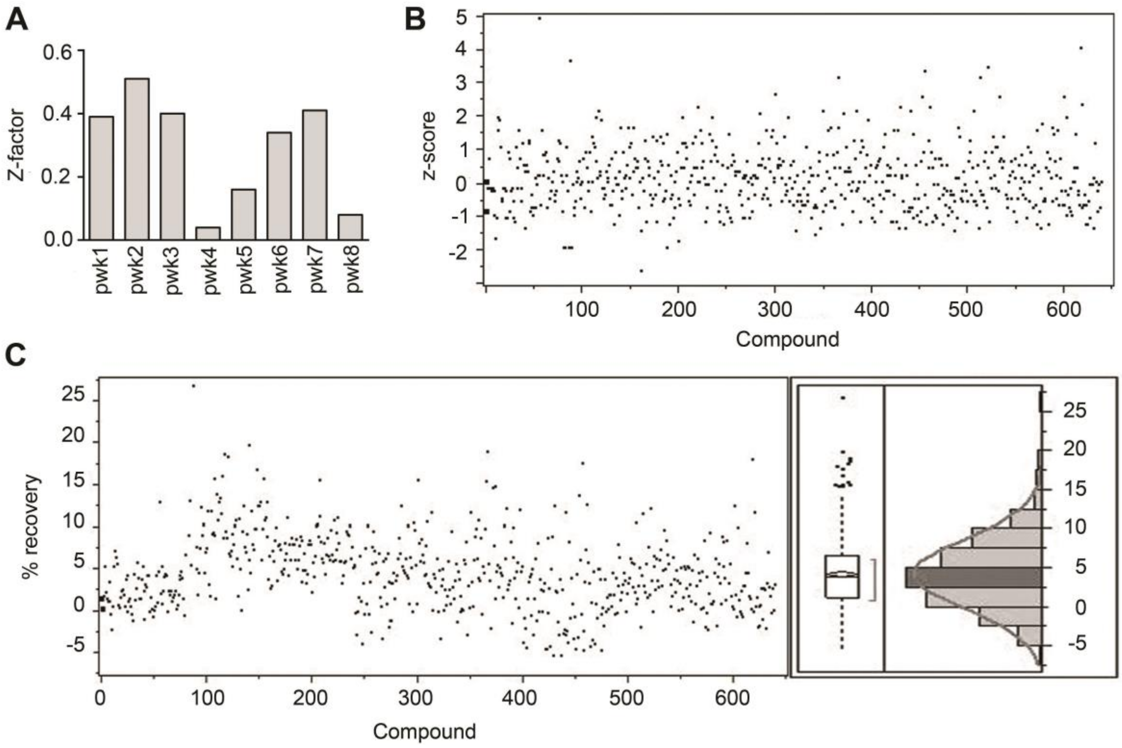

Supplementary Figure 3. Assessment of statistical screening parameters for the pilot *in vivo* spliceosensor screening method. (**a**) Z-factors obtained for the 640 Prestwick compounds (eight plates) in the pilot screening, showing all positive values. For final screening purposes, any plate with a Z-factor lower than 0 was determined to be screened again. (**b**) Scatterplot, outlier boxes and distribution of the percentage of recovery obtained with the 640 compounds assayed in the pilot screening. Graphs show normal distribution with mean around 0, normality was confirmed with a W=0.97 in a Shapiro-Wilk test.

SUPPLEMENTARY FIGURE 4

A

VLT037

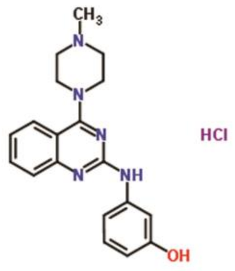

3-{[4-(4-Methyl-1-piperazinyl)-2-quinazolinyl]amino}phenol hydrochloride (1:1)

B

VLT027

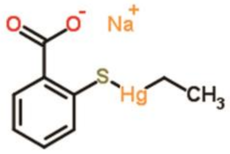

Sodium ethyl[2-(sulfanyl-κS)benzoato(2-)]mercurate(1-)

Supplementary Figure 4. Examples of molecules identified in the *in vivo* screening. Complete chemical name, along with their structures are shown for some confirmed hits: (**A**) VLT037 and (**B**) VLT027.

Supplementary Table 1. Chromosomal and genetic location of spliceosensor transformants.

**Transformant**	**Chromosome**[a]	**Genetic location** [b]	**Intergenic** [c]
UAS-INSR#1	3	3L:9.056.056	No (CG32031)
UAS-INSR#2	3	3R: 19.606.437	No (CG10198)
UAS.INSR#3	3	3L:7.361.936	No (CG8582)
UAS-INSR#4	3	3L:14.983.339	Yes
UAS-INSR#5	3	3R:21.717.153	Yes
UAS-INSR#6	3	3L:3.250.494	Yes
UAS-INSR#7	2	2L:14.614.224	No (CG3479)
UAS-INSR#8	1(X)	X: 8.318.048	No (CG18009)
UAS-cTNT#1	2	2L: 267.519	No (CG3645)
UAS-cTNT#2	2	2R: 9.915.017	No (CG8118)
UAS-cTNT#3	NA	NA	NA
UAS-cTNT#4	2	2L: 8.416.700	No (CG13398)
UAS-cTNT#5	NA	NA	NA
UAS-cTNT#6	3	3L:3.250.494	No (CG12078)
UAS-cTNT#7	3	3L: 3.250.522	Yes
UAS-cTNT#8	2	2L: 7.010.351	Yes
UAS-cTNT#9	NA	NA	NA
UAS-cTNT#10	3	3L: 8.818.598	No (CG4974)
UAS-TnnT3#1	3	3R:21.155.027	Yes
UAS-TnnT3#2	3	NA	NA
UAS-TnnT3#3	2	2L:825.813	Yes
UAS-TnnT3#4	3	3R:21.862.588	Yes
UAS-TnnT3#5	2	2R:14.499.381	Yes
UAS-TnnT3#6	3	3L:11.580.258	No (CG6097)
UAS-TnnT3#7	2	2R:13.680.196	Yes
UAS-TnnT3#8	1(X)	X:7.825.401	No (CG10777)
UAS-TnnT3#9	2	2L:18951814	No (CG10679)
UAS-TnnT3#10	3	3R:25.625.351	No(CG7788)

[a] Indicates genetic mapping results. [b] Shows reverse PCR results. [c] Whenever a transgene was inserted in a known gene, gene ID appears in brackets. *Drosophila* genome version used was r5.16.

Supplementary Table 2. Primers used for site-directed mutagenesis and for RT-PCR.

Primer	Secuence 5'-3'	Ta (Cycles)
clon luc F	AGCCACCCTCGAGGAAGACGCCA	62 (39)
clon luc R	GGGGTACCTTACACGGCGATCTTTCCGCCCTTCTTGG	
clon TnnT3 F	GGAATTCACCATGGGAGGAAGTCCAAGAAGGTAGGTG	59 (39)
clon TnnT3 R	TGCGCTCGAGTTGGGTCTTGGTTTCTCCTCTGGTCATG	
clon cTNT F	GGAATTCACCATGGCCGGTTCACAACCATCTAAAGC	58 (39)
clon cTNT R	CCCTCGAGGGCTACAAGATTGCTGGAGC	
clon INSR F	GGAATTCACCATGGGGAATGCTGCTCCTGTCCAAAGACAGACTCTCAGATCCTCCGAAGGAGCTG	65 (39)
clon INSR R	TTCCTCGAGCGTGGGCACGCTGGTCGAGGAAG	
INSR RT-PCR F	ACGTTTGAGGATTACCTGCACAA	60 (29)
INSR RT-PCR R	GAGATGGCCTGGAACGACAG	

Simple underlined indicates restriction sites added to the sequence. Double underlined indicates Kozak sequence for translation initiation. Bold underlined indicated nucleotides added to modify amino acid sequence.
